# Secondary Imaging Architecture for Fast and Ultra-Wide LWIR Optics with Low Rectilinear Distortion

**DOI:** 10.3390/s26082334

**Published:** 2026-04-09

**Authors:** Kuo-Chuan Wang, Cheng-Huan Chen

**Affiliations:** Department of Photonics, College of Electrical and Computer Engineering, National Yang Ming Chiao Tung University, 1001 University Road, Hsinchu 300, Taiwan

**Keywords:** LWIR, LEO, rectilinear, secondary imaging, curved intermediate image, chalcogenide glass

## Abstract

Wide-swath longwave infrared (LWIR) imaging from Low Earth Orbit (LEO) demands fast optics and rectilinear (F-tan) mapping for thermal mapping and multi-frame registration. Achieving an F/1.2 aperture with a 112° diagonal field of view (FOV) and distortion within ±5% is challenging, as mapping constraints and field-dominant off-axis aberrations become strongly coupled at large chief-ray angles. The low-distortion target is not only a geometric specification, but also a practical requirement that reduces peripheral compression, helps maintain edge-detail consistency, and lowers digital de-warping effort in the processing pipeline. While traditional LWIR secondary imaging is predominantly restricted to narrow-field cooled systems for cold-stop constraints, the proposed architecture utilizes a curved intermediate image to effectively decouple mapping formation in the field-dominant front objective from aperture-dominant correction in the rear group. Using chalcogenide glasses, the lens achieves a 5.7 mm effective focal length within a 186.9 mm total track. Analysis over the 8–12 μm band confirms performance approaching the diffraction limit at the 50 lp/mm Nyquist frequency alongside stable geometric fidelity across the full field. Thermal analysis from −40 °C to 80 °C and Monte Carlo tolerance analysis demonstrate stable imaging performance and manufacturing feasibility, confirming the effectiveness of the proposed design approach.

## 1. Introduction

Thermal infrared imaging in the 8–12 µm band enables passive day-night monitoring and is indispensable for spaceborne applications such as wildfire detection and Earth-radiation monitoring [1,2]. On LEO platforms, the demand for broad-swath coverage has intensified the development of wide-field longwave cameras, underscoring the system-level value of combining high optical throughput, radiometric consistency, and high geometric fidelity. In parallel, uncooled microbolometer focal plane arrays (FPAs) have matured significantly in pixel scaling and cost-effectiveness, making them increasingly attractive for small-satellite thermal payloads [3,4]. For space-like operation, long-term radiometric stability and calibration management are paramount to ensure product consistency over time [5]. These factors motivate optical designs that maximize information density per frame while minimizing artifacts or distortions that could hinder downstream analytics.

For LEO wide-area surveillance, geometric accuracy is as critical as spatial coverage. Mapping, multi-frame registration, and mosaicking pipelines benefit significantly from rectilinear (F-tan) projection, where the ideal mapping follows $y=ftan\left(\theta \right)$. In contrast, fisheye-style equidistant projection follows y=fθ, which redistributes angular scale and introduces strong barrel distortion relative to rectilinear geometry. As this distortion increases, peripheral content compression becomes severe, leading to reduced angular sampling density and lower feature richness near the field edges. Such degradation can significantly weaken the robustness of automated registration and feature-tracking algorithms used in wide-area analytics.

Prior ultra-wide-angle optical studies have commonly treated sub 5% distortion as a meaningful performance objective, although such reported targets or achieved values are generally based on the less stringent F-theta metric [6,7]. In the present work, the ±5% rectilinear (F-tan) criterion was therefore defined as a practical design threshold for the 112° diagonal field. Because rectilinear mapping follows $ftan\left(\theta \right)$ rather than fθ, this requirement imposes a more stringent edge-mapping constraint than the commonly reported F-theta-based criterion at the same field extent [8,9].

Achieving a fast aperture (e.g., F/1.2) and a super-wide field of view (FOV) exceeding 100° while maintaining rectilinear (F-tan) distortion below ±5% represents a significant optical challenge. A review of recent LWIR literature suggests that existing designs typically fall into two categories that do not fully satisfy high-fidelity space mapping requirements. First, many ultra-wide LWIR lenses utilize fisheye-type structures focused on equidistant projection, which are not directly compatible with standard mapping workflows [10,11]. Second, approaches attempting quasi-rectilinear behavior often accept substantial barrel distortion or rely on diffractive optical elements (DOE) with relatively high distortion bounds, which can hinder precision registration [12,13]. Furthermore, while many large-aperture LWIR systems exist, they often rely on hybrid or stitching-oriented architectures, or remain confined to narrower effective fields, reflecting the difficulty of balancing off-axis aberrations at large chief-ray angles [14,15].

Representative wide-field LWIR studies have also been reported in the literature, including a 140° F/1 wide-field camera and a 110° F/2 athermalized wide-angle lens [2,16]. Although these studies provide a useful broader context for ultra-wide LWIR imaging, their design emphases differ from those of the present work. In particular, the former accepts substantially larger distortion, whereas the latter employs a slower aperture than the present F/1.2 design. Moreover, their architectural bases and design objectives differ from those of the secondary-imaging framework investigated in this work.

The application of secondary imaging architectures in the LWIR band has been reported, yet these are often motivated by constraints different from ultra-wide mapping. As summarized in Table 1, current secondary imaging demonstrations provide evidence of the architecture’s versatility but predominantly serve narrow-field envelopes rather than the ultra-wide demands targeted in this work.

This comparison reveals a gap between prior secondary imaging LWIR designs and the combined target of F/1.2, a 112° diagonal FOV, and within ±5% rectilinear distortion. To address this gap, this work proposes an all-refractive two-group secondary imaging architecture and validates its effectiveness using group-wise third-order coefficient trends and full-field modulation transfer function (MTF).

The originality of this work lies in the architecture-level integration and validation of an all-refractive two-group secondary-imaging architecture for an ultra-wide, fast, low-distortion uncooled LWIR mapping system, rather than claiming secondary imaging itself to be introduced here for the first time.

This work is organized as follows: Section 2 establishes the mapping metrics and the conceptual framework of secondary imaging. Section 3 details the optical configuration, staged optimization procedure and the results of simulations, including thermal stability evaluations and Monte Carlo tolerance analysis. Section 4 provides a comprehensive results analysis based on third-order aberration coefficients. Finally, Section 5 concludes the work.

## 2. Concept of Secondary Imaging

This section establishes the analytical framework linking optical resolution to the digital sampling limit and explores the first-order framework required to decouple rectilinear mapping from fast-aperture aberrations.

Based on the detector parameters described previously, the Nyquist spatial frequency $f_{N}$, which defines the upper limit of spatial sampling [20,21], is given by:

(1) $$f_{N}=\frac{1}{2p},$$

where p is the detector pixel pitch. For a 10 μm pitch, $f_{N}$ = 50 lp/mm. Optical performance is evaluated using the MTF at this frequency, providing a metric directly linked to the detector’s spatial sampling capacity and the detail required for downstream feature extraction [21,22].

**Figure 1:**
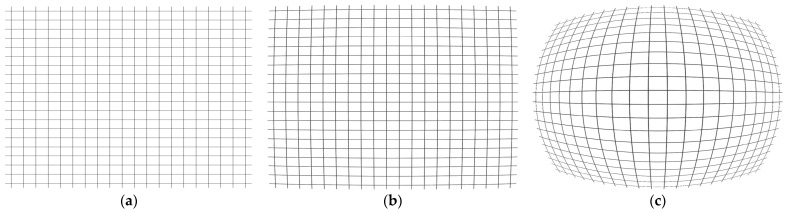
Grid deformation illustration with 112° diagonal FOV. (**a**) Ideal rectilinear mapping $y_{ideal}\left(\theta \right)=f\tan\left(\theta \right)$ (no distortion); (**b**) −5% rectilinear (F-tan) distortion at the corner (barrel) relative to the rectilinear ideal; (**c**) equidistant (F-theta) projection shown for reference with a −5% radial scaling error at the corner relative to the F-theta ideal $y_{ideal}\left(\theta \right)=f\theta$.

Geometric fidelity is assessed using a rectilinear (F-tan) projection model, where the ideal mapping between the field angle θ and image height $y_{ideal}\left(\theta \right)$ is given by [23,24]:

(2) $$y_{ideal}\left(\theta \right)=f\tan\theta ,$$

where f is the effective focal length (EFL). Accordingly, the rectilinear distortion is quantified as the relative radial deviation from this ideal tangent profile:

(3) $$Distortion\left(\theta \right)=\frac{y\left(\theta \right)-y_{ideal}\left(\theta \right)}{y_{ideal}\left(\theta \right)}\times 100\%,$$

where $y\left(\theta \right)$ is the actual image height obtained from ray tracing. Constraining this metric across the field directly limits peripheral content compression, preserving the information density near the field edge and reducing the computational burden of digital de-warping. Figure 1 provides a visual comparison between the ideal rectilinear mapping, the targeted −5% barrel distortion limit, and the standard equidistant projection.

Based on these definitions, two fundamental first-order relations explain why super-wide rectilinear FOV, fast aperture, and low rectilinear (F-tan) distortion are tightly coupled. First, the Lagrange invariant H is defined as [24,25]:

(4) H=nysinu≈nyu,

where n is the refractive index, y is the ray height at a given pupil, and u is the marginal-ray angle. In a traditional single-stage ultra-wide lens, the front elements must handle the maximum field height y while simultaneously accommodating a fast F-number cone (u is large). This simultaneous increase in both y and u at the same physical surface generates massive aberration residuals that are extremely difficult to balance. Secondary imaging provides a mechanism for spatial decoupling: Group 1 (Gr1) manages the large chief-ray angles required for wide field coverage at a lower aperture, while Group 2 (Gr2) performs high-speed aperture correction at the final stage.

Second, the mapping can be expressed in a distortion-series form [23,24,25]:

(5) $$y\left(\theta \right)=y_{ideal}\left(\theta \right)[1+k_{1}\tan^{2}\left(\theta \right)+k_{2}\tan^{4}\left(\theta \right)+\cdots ].$$

Substituting Equation (5) into Equation (3) yields the rectilinear (F-tan) distortion approximation:

(6) $$\mathrm{Distortion}\left(\theta \right)\approx [k_{1}\tan^{2}\left(\theta \right)+k_{2}\tan^{4}\left(\theta \right)+\cdots ]\times 100\%.$$

In a centered rotationally symmetric system, the departure from the rectilinear (F-tan) mapping is naturally expressed as an even-power series in tan(θ). Equation (5) therefore lists the leading nonzero correction terms explicitly to highlight the order structure of the distortion expansion, while the omitted higher-order remainder is retained in the ellipsis. In the present super-wide design, $k_{1}$ captures the dominant third-order mapping trend, whereas $k_{2}$ and higher-order terms mainly refine the residual distortion terms.

**Figure 2:**
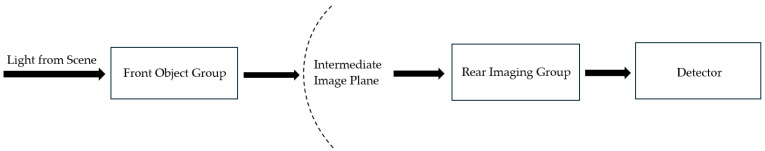
Secondary imaging concept.

The secondary imaging architecture facilitates this separation of aberration correction tasks by forming a curved intermediate image, as illustrated in Figure 2. The curved intermediate image surface plays a crucial role in separating field-dominant mapping formation from high-aperture aberration correction. By intentionally allowing the intermediate image to remain curved rather than forcing it to be flat at the first stage, the design avoids spending the limited degrees of freedom in Gr1 on premature field flattening. This non-flat intermediate state acts as an architectural buffer that Gr1 can concentrate on establishing the required rectilinear mapping trend, whereas Gr2 subsequently re-images the curved intermediate image onto the flat detector and completes the final field-flattening together with aperture-dominant residual correction. The merit of this strategy therefore lies not in suppressing distortion within the front group itself, but in allowing the dominant rectilinear mapping trend to be generated in a controlled first stage, while deferring the final field-flattening and fast-aperture residual correction burden to Gr2.

## 3. Optical Design and Simulation

The optical model was implemented and optimized using CODE V, version 2025.03 (Synopsys, Sunnyvale, CA, USA) [26] based on the specifications in Table 2, utilizing weighting across the 8–12 µm band and strategic field sampling to cover the full diagonal FOV, ensuring comprehensive coverage for the whole detector format. The optimization prioritized the metrics defined in Section 2: minimizing rectilinear (F-tan) mapping error and maximizing MTF at the 50 lp/mm Nyquist limit. Practical mechanical constraints, including total track length and the limited glass set of SCHOTT IRG22–IRG27 infrared glasses (SCHOTT AG, Mainz, Germany), were enforced throughout the optimization process [27].

**Figure 3:**
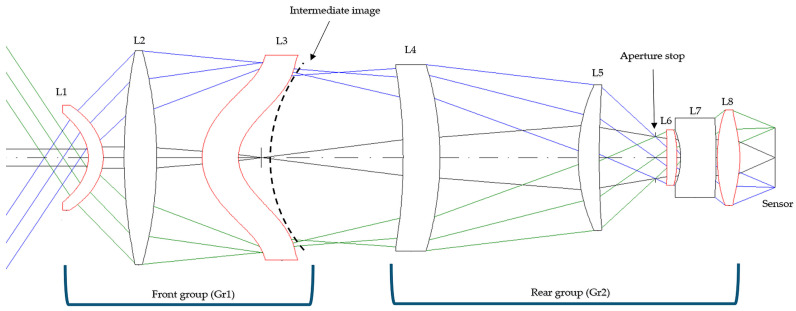
Final optimized optical layout of the proposed two-group eight-element LWIR lens. This sectional layout was extracted from the nominal CODE V model, with labels added for clarity. Colored rays are shown only to distinguish ray bundles corresponding to different fields in the optical layout. Gr1 (L1–L3) forms the curved intermediate image, and Gr2 (L4–L8) re-images it onto the detector. The aperture stop is located between L5 and L6. Both surfaces of L1, L3, L6, and L8 are aspheric.

The system adopts the two-group configuration illustrated in Figure 3, where Gr1 (L1–L3) generates the intermediate image. The aperture stop is positioned between L5 and L6 within the rear group (Gr2) to facilitate final-stage correction at the detector plane.

Table 3 summarizes the element focal lengths, clear apertures, and materials from the final optimized model. Schott IRG-series chalcogenide glasses (Table 3) were selected for their refractive index and dispersion balance, with IRG24 serving as the primary material for most elements.

To achieve high-order correction within an eight-element constraint, aspheric surfaces were strategically positioned on L1, L3, L6, and L8, resulting in a total of eight aspheric surfaces in the final design. Aspheres in Gr1 manage wide-angle aberrations and establish the initial mapping profile, whereas those in Gr2 refine aperture-dominant residuals and perform final-stage distortion balancing under the F/1.2 cone. Manufacturability was assessed within standard fabrication and metrology limits.

The comparatively large TTL/EFL ratio reflects a system-level tradeoff made to satisfy the combined requirements of a 112° diagonal FOV, F/1.2 aperture, and rectilinear (F-tan) distortion within ±5%. In this design, optical power is intentionally distributed over a longer track to avoid excessive surface power concentration, which would otherwise increase sensitivity to fabrication and assembly errors and make residual aberration balancing more difficult. A shorter package may be achievable with stronger element powers, more aggressive aspheric departures, or folded configurations, but such approaches could tighten tolerance margins and compromise the present balance among distortion control, imaging performance, and manufacturability. The retained 186.9 mm total track should therefore be regarded as a deliberate engineering tradeoff rather than an inefficiency.

The optimization was conducted in two distinct stages to decouple field-mapping constraints from aperture-dominant corrections:

1. Stage A (Gr1 mapping formation): This stage prioritizes geometric constraints to enforce the rectilinear (F-tan) profile and ensure stable intermediate image formation.
2. Stage B (Gr2 aperture-dominant correction): With the intermediate image established, weights on wavefront error and MTF (at 50 lp/mm) were increased. This stage focused on correcting residuals under the stop-controlled F/1.2 cone.

**Figure 4:**
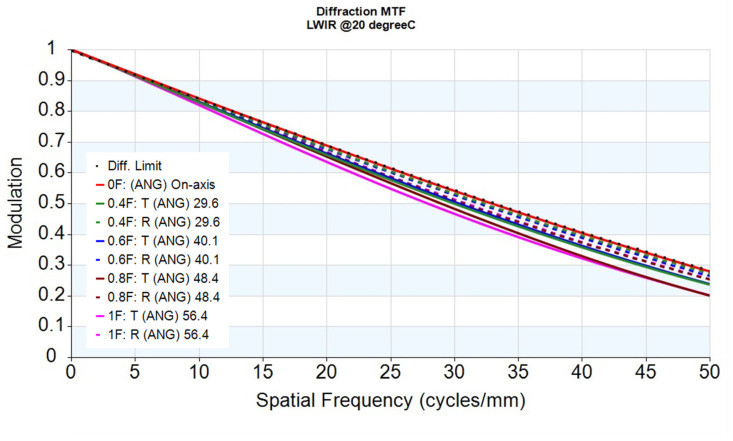
MTF at 20 °C with the diffraction-limited reference.

Regarding nominal imaging performance, Figure 4 presents the MTF at 20 °C, which closely tracks the diffraction-limited reference across all sampled fields. This indicates good image quality, with the MTF at 50 lp/mm maintaining high modulation to support sampling-limited imaging.

**Figure 5:**
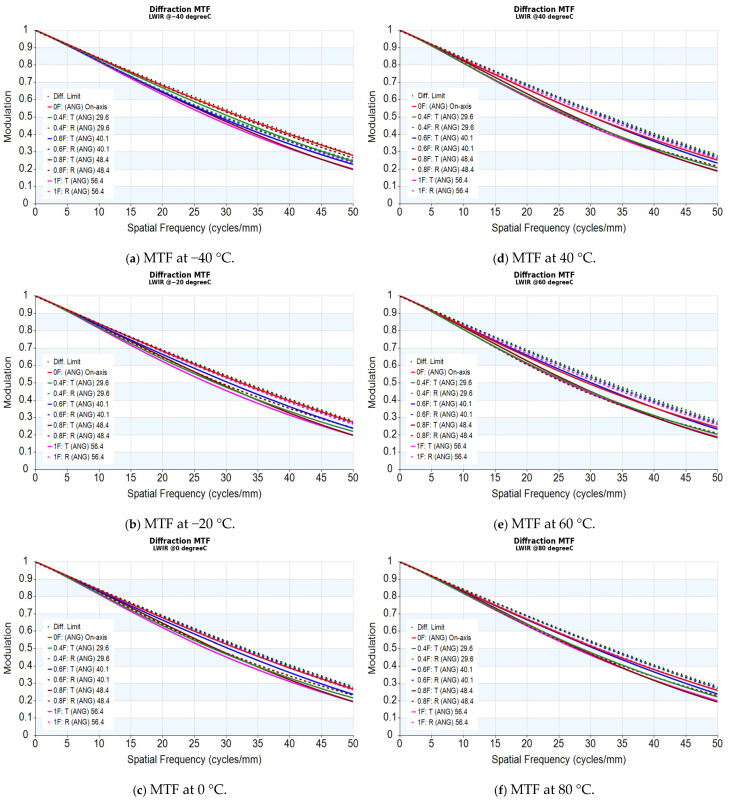
MTF performance under different operating temperatures.

Thermal stability was evaluated from −40 °C to 80 °C. As shown in Figure 5a–f, the MTF remains stable and near the diffraction limit across the entire temperature range. These results account for the combined effects of thermo-optic responses and structural expansions, confirming that the design retains its resilience without pronounced degradation. The thermal model utilizes an Invar 36 barrel and spacers, leveraging its ultra-low coefficient of thermal expansion (CTE) to effectively suppress thermomechanical change [28]. This choice of housing material ensures that Nyquist-relevant contrast is well preserved across the extreme temperature excursions typical of spaceborne operations.

The thermal stability shown in Figure 5 is further summarized in Table 4 by the spatial frequencies at which all analyzed fields remain above 15% and 50% MTF. Relative to the 20 °C baseline, the all-field 15% MTF frequency varies only from 53.5 to 54.6 lp/mm, and the all-field 50% MTF frequency varies from 26.3 to 27.6 lp/mm over the evaluated temperature range. These compact metrics provide a clearer comparison of thermal MTF shift.

**Figure 6:**
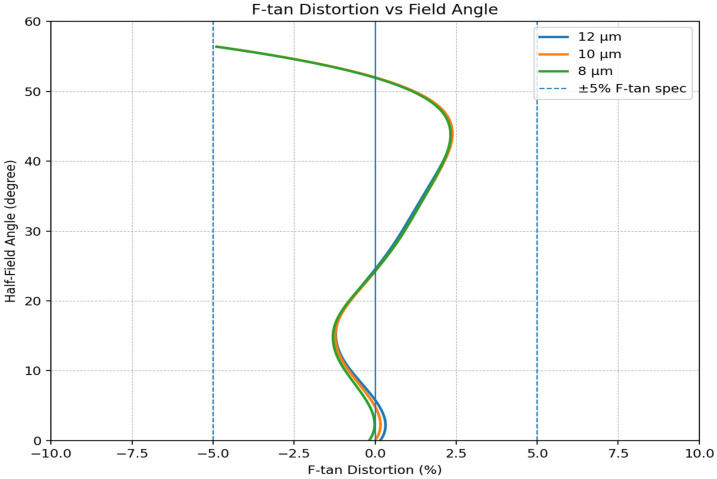
Rectilinear (F-tan) distortion vs. half-field angle.

Geometric fidelity is summarized in Figure 6, with rectilinear (F-tan) distortion remaining within ±5% over the full 112° diagonal field. For the selected 1280 × 1024 detector with a 10 μm pixel pitch, the peak distortion at the diagonal corner corresponds to an inward positional deviation of approximately 40 pixels relative to the ideal rectilinear image location. This quantity denotes cumulative geometric displacement, whereas edge-field compression refers to the reduction in pixels assigned to a given angular interval. In this sense, the present mapping better preserves edge sampling capacity than a conventionally compressed wide-field mapping trend.

**Figure 7:**
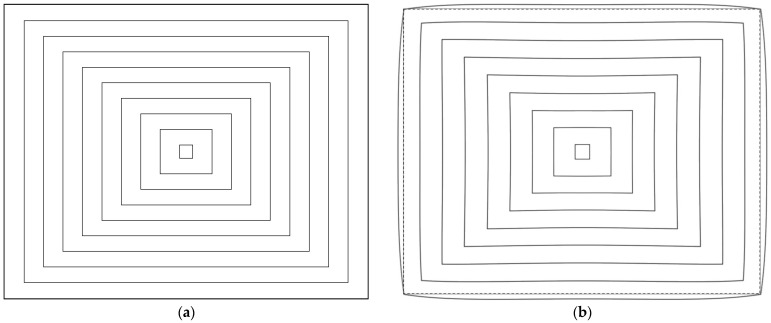
Detector-format 2D image simulation of the final optical design using a concentric rectangle target: (**a**) ideal input target and (**b**) simulated image of this work mapped onto the 1280 × 1024 sensor format. The dashed outer rectangle in (**b**) indicates the sensor active area.

To provide an image-domain visualization of the final mapping behavior, Figure 7 presents a detector-format 2D image simulation of the optimized optical design using a concentric rectangle target, generated with the 2D image simulation function in CODE V. Figure 7a shows the ideal input target, and Figure 7b shows the corresponding simulated image on the 1280 × 1024 sensor format. In this setup, the four corners of the ideal target were assigned to the full-field corners of the sensor. Although slight barrel-type deformation remains near the edge field, the simulated pattern indicates markedly reduced peripheral compression compared with the conventional F-theta-type mapping conceptually shown in Figure 1, which helps preserve edge information density for subsequent processing. This behavior is consistent with the distortion trend in Figure 6.

**Figure 8:**
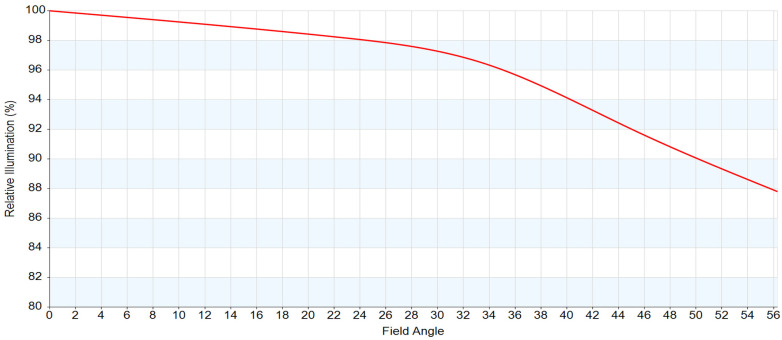
Relative illumination profile.

Additionally, relative illumination (RI) was evaluated to assess radiometric uniformity. As shown in Figure 8, the secondary imaging architecture manages pupil expansion and maintains RI above 80% at the corner field. This reduces the corner signal-to-noise (SNR) penalty in wide-swath operation. Note that standard non-uniformity correction (NUC) methods [29,30] mainly compensate for pixel-response nonuniformity and cannot fully recover optical illumination fall-off.

The manufacturing feasibility was further assessed through a 5000-trial Monte Carlo simulation based on the high-precision tolerance budget in Table 5. Parameters such as radius, thickness, and assembly misalignments were assigned according to rigorous fabrication standards.

**Figure 9:**
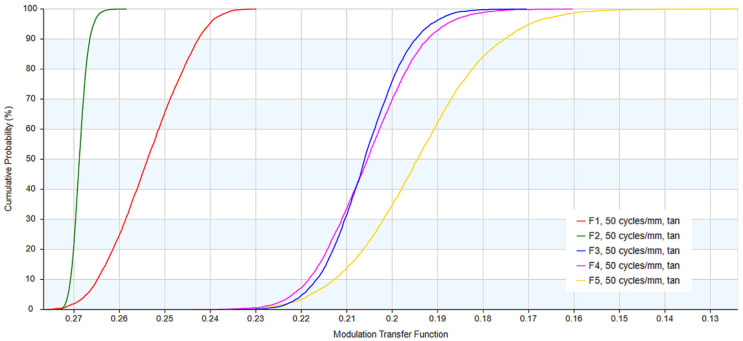
Cumulative probability distribution MTF at 50 lp/mm from a 5000-trial Monte Carlo simulation.

The cumulative probability distribution (Figure 9) suggests that the design is robust against production residuals across the sampled fields F1–F5. While the absolute MTF at 50 lp/mm is intrinsically limited by the F/1.2 diffraction envelope, the simulation indicates high performance stability. At a 90% confidence level, the system maintains an MTF above 0.15 across the entire FOV. The steepness and tight clustering of the probability curves for F1 (0°) through F5 (56°) indicate that secondary imaging architecture successfully desensitizes the system to fabrication errors. This level of contrast retention is considered sufficient for supporting high-fidelity thermal imaging and feature extraction in 10 µm-pitch uncooled LWIR payloads.

## 4. Results Analysis and Discussion

This section validates the internal balancing mechanism using element-wise third-order aberration coefficients extracted from the nominal model. To adhere to classical Seidel definitions, the analysis is restricted to intrinsic contributions from base spherical surfaces. By isolating these geometric residuals, the decoupling strategy proposed in Section 2, separating field-dominant mapping from aperture-dominant correction, can be quantitatively assessed.

The group-wise sums of these intrinsic third-order contributions are consolidated in Table 6. The raw surface-by-surface coefficients are listed in Appendix A Table A1; only the spherical entries are used for the Section 4 calculations. In this section, Spherical aberration (SA), Tangential coma (TCO), Distortion (DST), and Petzval sum (PTZ) denote third-order quantities directly extracted from the nominal CODE V aberration analysis. No additional normalization or hidden scaling was applied. These coefficients are used as diagnostic indicators to interpret group-wise task allocation and mapping tendency in the base spherical design. They are distinct from the normalized mapping-series formulation introduced in Section 2.

The contribution in Table 6 reveals a clear functional partition. First, the aperture-dominant correction task is isolated within Gr2, where the Spherical aberration (SA) is concentrated (0.344) compared to the negligible contribution of 0.008 from Gr1. This confirms that the rear group successfully manages the high-aperture (F/1.2) cone residuals. Second, the rectilinear mapping foundation is established primarily through the front group’s geometric layout, as evidenced by the massive Distortion (DST) of −21.014 generated in Gr1. Third, the total Petzval sum (PTZ) of −0.011 demonstrates that the base optical powers and materials are inherently balanced for field flatness.

To further illustrate the mapping formation and the cancelation of aperture-sensitive aberrations, Table 7 tracks the cumulative trends across the optical path. The accumulation trend in Table 7a shows that the rectilinear mapping is primarily shaped within Gr1 (L1–L3) through a succession of barrel-type contributions, while Gr2 provides minor stabilization. Table 7b highlights the compensatory balancing of SA and TCO within the rear group.

Section 4 therefore establishes a spherical third-order foundation for task allocation. Aspheric contributions are treated separately as implementation-level residual correctors as shown in Table A2. This supports a two-step strategy in which the base configuration decouples major aberration burdens, followed by final residual closure in the implementation stage.

## 5. Conclusions

This work presents an ultra-wide, fast-aperture secondary-imaging LWIR lens for LEO applications. The proposed architecture introduces a curved intermediate image and separates field-dominant mapping control in the front group from aperture-dominant aberration correction in the rear group. The final eight-element all-refractive design achieves F/1.2, a 112° diagonal FOV, and rectilinear (F-tan) distortion within ±5% over 8–12 μm for a 1280 (H) × 1024 (V) uncooled microbolometer with a 10 μm pixel pitch. At the Nyquist frequency of 50 lp/mm, the nominal MTF approaches the diffraction-limited trend. Beyond meeting geometric specifications, the low-distortion design also reduces peripheral compression and de-warping effort, which supports more stable edge-detail usage in downstream processing.

Furthermore, third-order coefficient analysis based on spherical-surface terms confirms the intended front-to-rear task partition. Thermal evaluation from −40 °C to 80 °C and a 5000-trial Monte Carlo tolerance analysis further support stable imaging performance and manufacturability. These results support the proposed secondary-imaging strategy for spaceborne LWIR thermal-mapping payloads that require high throughput, rectilinear mapping fidelity, and environmental robustness.

## Figures and Tables

**Table 1:** Representative secondary imaging designs for LWIR in published literature and proposed specification of this work.

Category (Typical Goal)	Representative Work	Band (μm)	F/#	FOV	Notes Relevant to This Work
Cooled system (pupil/cold-stop)	Sun et al. (Dyson spectrometer) [17]	8–12	2	2.93°	Secondary imaging used for cooled spectrometer and cold-stop constraints; very narrow FOV
Narrow-FOV	Zhang et al. (F/1.0 LWIR) [13]	8–12	1	4°	Narrow FOV; focuses on secondary imaging form and stray-light/layout considerations rather than rectilinear ultra-wide distortion control
Cooled multi-channel (common-aperture catadioptric telescope)	Zhang et al. (three-channel common-aperture RC telescope) [18]	7–11	2	4°	Cooled detector; cold-stop constrained; narrow-FOV telescope-type; not aimed at rectilinear ultra-wide mapping
Variable-FOV (mast/periscope payload)	Raju et al. (FOV-changing) [19]	7.5–9.5	1.95	4.15°/12.45°	Secondary imaging architecture supports magnification/FOV switching; still far from >100° rectilinear
This work (uncooled mapping-oriented)	All-refractive secondary imaging	8–12	1.2	112° (diagonal)	Rectilinear mapping (F-tan) within ±5% distortion; uncooled-compatible; manufacturable glass set

**Table 2:** Optical specifications of LWIR lens module.

Items	Symbol	Value	Unit	Description/Note
Sensor Resolution		1280 × 1024	—	Horizontal × Vertical
Sensor Pixel Size		10	μm	
Working wavelength		8–12	μm	Longwave infrared
Effective Focal Length	f	5.7	mm	System EFL
F-number	F/#	1.2	—	
Field of View	D-FOV	112	°	Diagonal field of view
Total Track Length	TTL	186.9	mm	First surface to image plane
Back Focal Length	BFL	9.6	mm	Last surface to image plane
Optical Distortion		within ±5	%	Rectilinear (F-tan)

**Table 3:** Element-level focal length, clear aperture, and material.

Element No.	Element EFL (mm)	Clear Aperture (mm)	Materials (Schott)
1	315.12	28.77	IRG24
2	39.97	58.54	IRG24
3	−475.15	52.07	IRG22
4	139.03	51.03	IRG27
5	35.47	40.02	IRG24
6	24.75	15.18	IRG24
7	−15.44	21.74	IRG27
8	12.27	26.09	IRG24

Note: Element EFLs are reported at the nominal wavelength of 10 μm at 20 °C.

**Table 4:** Summary of the spatial frequencies for which all analyzed fields maintain MTF values above 15% and 50% across the evaluated temperature range.

Temperature (°C)	MTF > 15% Frequency (lp/mm)	MTF > 50% Frequency (lp/mm)
80	54.5	27.6
60	53.5	26.3
40	54.1	26.5
20	54.2	27.0
0	54.3	27.0
−20	54.5	27.2
−40	54.6	27.5

**Table 5:** Tolerance budget for precision manufacturing standards.

Parameter	Tolerance Value	Unit
Radius (Fringe/Irregularity)	3/1	fringes
Thickness	±0.015	mm
Lens decenter	±0.01	mm
Lens tilt	±1	arcmin
Assembly tilt (Element)	±1	arcmin
Material index	±0.0005	—
Material dispersion	±0.5	%

**Table 6:** Group-wise sums of spherical third-order contributions.

Group	SA (mm)	TCO (mm)	DST (mm)	PTZ (mm^−1^)
Gr1 (L1–L3)	0.008	−0.178	−21.014	0.024
Gr2 (L4–L8)	0.344	−0.301	0.228	−0.035
Total (Base Sphere)	0.352	−0.479	−20.786	−0.011

**Table 7:** Cumulative coefficient trends illustrating cancelation.

(**a**) Cumulative DST Trend Across L1–L8 (mm).				
Lens	DST		Cumulative DST	
1	−7.988		−7.988	
2	−7.139		−15.127	
3	−5.887		−21.014	
4	0.340		−20.674	
5	1.788		−18.886	
6	−0.695		−19.581	
7	1.125		−18.456	
8	−2.330		−20.786	
(**b**) SA and TCO Cancelation Within the Rear Group (from L4 to L8) (mm).				
Lens	SA	TCO	Cumulative SA	Cumulative TCO
4	0.005	−0.035	0.005	−0.035
5	0.101	0.220	0.106	0.185
6	0.443	1.120	0.549	1.305
7	−0.736	−1.260	−0.187	0.045
8	0.531	−0.346	0.344	−0.301

## Data Availability

The data presented in this study are available on request from the corresponding author.

## Abbreviations

The following abbreviations are used in this manuscript:

BFL	Back focal length
DOE	Diffractive optical element
DST	Third-order tangential distortion
EFL	Effective focal length
FOV	Field of view
FPA	Focal plane array
Gr1	Front objective group
Gr2	Rear group
LEO	Low Earth Orbit
MTF	Modulation transfer function
NUC	Non-uniformity correction
PTZ	Petzval sum
RI	Relative illumination
SA	Spherical aberration
SNR	Signal-to-noise
TCO	Tangential coma
TTL	Total track length

## Appendix A

This appendix separates surface aberration contributions into third-order spherical and aspheric components for clarity and traceability. Table A1 summarizes the spherical-surface contributions and is the sole input to the formal third-order calculations presented in Section 4. Table A2 lists the aspheric-surface contributions, which are used for implementation-level residual correction and post-optimization interpretation.

**Table A1:** Spherical-surface contributions to aberration coefficients.

Element	Surface	SA (mm)	TCO (mm)	DST (mm)	PTZ (mm^−1^)
L1	1	−0.008	0.168	8.968	0.059
	2	0.013	−0.352	−16.956	−0.049
L2	3	0.000	0.000	−6.096	−0.004
	4	0.003	−0.048	−1.043	−0.006
L3	5	0.000	0.024	12.058	−0.038
	6	0.000	0.030	−17.945	0.062
L4	7	0.005	−0.047	0.023	0.004
	8	0.000	0.012	0.317	−0.007
L5	9	0.079	−0.075	−0.065	−0.013
	10	0.022	0.295	1.853	0.002
L6	12	−0.017	−0.224	−1.324	−0.002
	13	0.460	1.344	0.629	−0.014
L7	14	−0.737	−1.302	−0.423	0.030
	15	0.001	0.042	1.548	−0.002
L8	16	0.000	0.015	−2.259	−0.014
	17	0.531	−0.361	−0.071	−0.019
SUM		0.352	−0.479	−20.786	−0.011

**Table A2:** Aberrations of aspheric-surface contributions.

Element	Surface	SA (mm)	TCO (mm)	DST (mm)	PTZ (mm^−1^)
L1	1	0.105	−4.236	−256.302	—
	2	−0.105	4.477	303.576	—
L3	5	−0.002	0.143	34.943	—
	6	0.001	−0.127	−57.911	—
L6	12	−0.875	1.002	0.049	—
	13	0.601	−0.945	−0.086	—
L8	16	−0.089	0.383	0.262	—
	17	−0.023	0.132	0.159	—
SUM		−0.387	0.829	24.690	—
